# Estrogenic Compounds or Adiponectin Inhibit Cyclic AMP Response to Human Luteinizing Hormone in Mouse Leydig Tumor Cells

**DOI:** 10.3390/biology8020045

**Published:** 2019-06-11

**Authors:** Thi Mong Diep Nguyen, Danièle Klett, Yves Combarnous

**Affiliations:** 1 Physiologie de la Reproduction et des Comportements, Institut National de la Recherche Agronomique (INRA), Centre National de la Recherche Scientifique (CNRS), 37380 Nouzilly, France; diepdhqn@ymail.com (T.M.D.N.); daniele.klett@inra.fr (D.K.); 2 Quy Nhon University, Quy Nhơn, Vietnam

**Keywords:** adiponectin, testosterone, progesterone, estradiol, bisphenol-A

## Abstract

Mouse Leydig Tumor cells (mLTC), transiently expressing cAMP-dependent luciferase, were used to study the influence of sexual steroids and of adiponectin (ADPN) on the cAMP response to luteinizing hormones (LH). While testosterone and progesterone had no significant effect, several molecules with estrogenic activity (17β-estradiol, ethynylestradiol, and bisphenol A) provoked a decrease in intracellular cyclic AMP accumulation under 0.7 nM human LH stimulation. Adiponectin exhibited a bimodal dose-effect on LH response: synergistic between 2–125 ng/mL and inhibitory between 0.5–5 µg/mL. In brief, our data indicate that estrogens and ADPN separately exert rapid (<1 h) inhibitory and/or synergistic effects on cAMP response to LH in mLTC-1 cells. As the inhibitory effect of each estrogenic molecule was observed after only 1-h preincubation, it might be mediated through the G protein-coupled estrogen receptor (GPER) membrane receptor, but this remains to be demonstrated. The synergistic effect with low concentrations of ADPN with human Luteinizing Hormone (hLH) was observed with both fresh and frozen/thawed ADPN. In contrast, the inhibitory effect with high concentrations of ADPN was lost with frozen/thawed ADPN, suggesting deterioration of its polymeric structure.

## 1. Introduction

Leydig cells play an essential role in testicular functions and male physiology. They respond to luteinizing hormones (LH) with an increase in intracellular cyclic AMP and subsequently produce testosterone that exerts feedback control on the pituitary and stimulates male characters (behavior, muscle mass) and the development and function of accessory glands (prostate, seminal vesicle, deferens duct) [1]. Testosterone from Leydig cells also strongly influences gametogenesis through its action into Sertoli cells [2]. Mouse Leydig Tumor Cells (mLTC) are a cell line retaining many of the testicular Leydig cells’ characteristics, except their steroidogenesis that almost stops at the level of progesterone production and synthesizes only small quantities of testosterone [3].

Leydig cells are targets for testosterone [4] and also for other steroid hormones [5] and, thus, we tested whether the cAMP response of mLTC cells to a submaximally stimulating concentration of human LH (0.7 nM), could be altered by testosterone or estrogens.

Adiponectin (ADPN) is a hormone involved in the control of metabolism and insulin sensitization. It is produced by mature adipocytes [6] and exhibits pleiotropic properties. It acts through two adiponectin receptors (AdipoR1 and AdipoR2), which are expressed in many cell types [7]. Besides, the expression of ADPN gene and protein has also been found in the reproductive system. It was noted, among others, in the hypothalamus [8] in the human endometrium, as well as the uterus, trophoblasts, and conceptuses of mice and pigs [9,10,11]. ADPN possesses anti-inflammatory properties for many tissues [12,13], as well as antiatherogenic and anticancerogenic properties [14,15,16]. It is mostly known for its role in the liver where it inhibits gluconeogenesis and stimulates glucose transport, oxidation of fatty acids, and insulin sensitivity [17], the latter also improving insulin receptor phosphorylation [18].

Gonadotropins are heterodimeric glycoprotein hormones acting through specific G Protein-Coupled Receptors (GPCR) to stimulate their target cells where 3’5’ cyclic adenosine monophosphate (cAMP) is their main intracellular second messenger. To study the intracellular signaling downstream of Luteinizing Hormone (LH) binding to LH receptor (LHR), we made use of the mLTC cell line in which we transiently expressed a cAMP-dependent luciferase. The mLTC-1 cell line has been established many years ago [3] and has been found of great interest for the study of Leydig cell sensitivity to LHs and human Chorionic Gonadotropin (hCG) in terms of binding or cAMP accumulation and steroid synthesis [19].

Steroid hormones and peptide hormones generally act via distinct mechanisms, the former most often via intracellular receptors through genomic activation [20] and the latter via membrane-localized receptors, such as the LHR, which stimulates the generation of intracellular cAMP. The cAMP-dependent kinase (PKA (protein kinase A)) and other protein kinases have also been documented to synergize with steroid hormone-occupied receptors, leading to enhanced steroid receptor-mediated transcription [21,22,23], possibly by a mechanism involving phosphorylation of the receptor or associated transcription factors [24,25,26]. In the present work, we show that estrogenic molecules, as well as ADPN, modulate human Luteinizing Hormone (hLH)-stimulated cyclic AMP accumulation in mLTC-1 cells.

## 2. Materials and Methods

### 2.1. Hormones

Recombinant human LH (hLH-C35) was from Serono (Geneva, Switzerland). Recombinant bovine adiponectin was purchased from Cusabio (Interchim, Montluçon, France). Steroid hormones were purchased from Sigma–Aldrich (Saint Quentin Fallavier, France).

### 2.2. Chemicals

All other chemicals were purchased from Sigma–Aldrich (Saint Quentin Fallavier, France) unless otherwise noted. pGlosensor–TM-22F cyclic AMP plasmid and CellTiter-Blue Cell viability assay (G8080) were from Promega (Charbonnières-les-Bains, France). XtremeGENE HP DNA transfection reagent was from Roche (Boulogne-Billancourt, France).

### 2.3. Cell Culture

The mLTC-1 cells [3] were obtained from the American Tissue and Cell Collection (ATCC) (LGC Standards, Molsheim, France). Cells were expanded in supplemented RPMI-1640 medium (Gibco, Invitrogen, 10% fetal bovine serum, 50 µg/mL gentamicin, 10 units of penicillin/mL, and 10µg/mL streptomycin). All cells were grown at 37 °C and 5% CO_2_. They were used between their 5th and 30th passage, a range in which they exhibited no decrease in their responses to LH.

### 2.4. Plasmids, Transfections

Cells (about 100,000 cells per well) on a 96-well Greiner white/clear bottom plate (Dutscher, Brumath, France) were transfected with pGlosensor-TM-22F cyclic AMP plasmid using XtremeGENE HP DNA transfection reagent. Thirty minutes before transfection, DNA (100 ng plasmid per well) and XtremeGENE HP DNA transfection reagent (0.3 µL per well) were mixed together with serum-free RPMI medium. This plasmid consists of firefly luciferase sequence fused to that of the PKA cAMP-binding domain in a way that allows control of its enzymatic activity by cyclic AMP. The plates were then incubated overnight at 37 °C under 5% CO_2_ before use in the assays.

### 2.5. cAMP Quantitation

Transfection supernatants were removed and replaced with a medium deprived of fetal-calf serum (100 µL) and containing the luciferase substrate luciferin and also 1 mM isobutyl-methyl-xanthine (IBMX) in order to inhibit endogenous nucleotide phosphodiesterase (PDE) activity. The plates were incubated for 1 h, before the addition of ADPN, testosterone, progesterone, 17β-estradiol (E2β), ethynylestradiol (EE2), or bisphenol A (BPA) at various concentrations in a 10 µL-volume. The cells were then incubated for another hour and, finally, human LH was added in a 10 µL volume in triplicate or sextuplicate wells to reach 0.7 nM concentration. Kinetics of intracellular oxyluciferin luminescence was then recorded using a Polarstar Optima (BMG Labtech Sarl, Champigny-sur-Marne, France) luminometer.

### 2.6. Area Under Curve (AUC) Calculations and Statistical Analyses

Slope calculations by linear fitting of initial accumulation rate and statistical analyses were carried out with GraphPad Prism 5 software (GraphPad Software, San Diego, CA, USA). The slope values are expressed as means ± SD of three independent experiments. For all single comparisons between each dose of sample and control, one-tailed paired Student’s t-test was performed after checking for distribution normality. For all statistical analyses, *p* < 0.05 was considered significant.

## 3. Results

### 3.1. Effects of Steroid Hormones on LH-Stimulated cAMP Response

**Figure 1:**
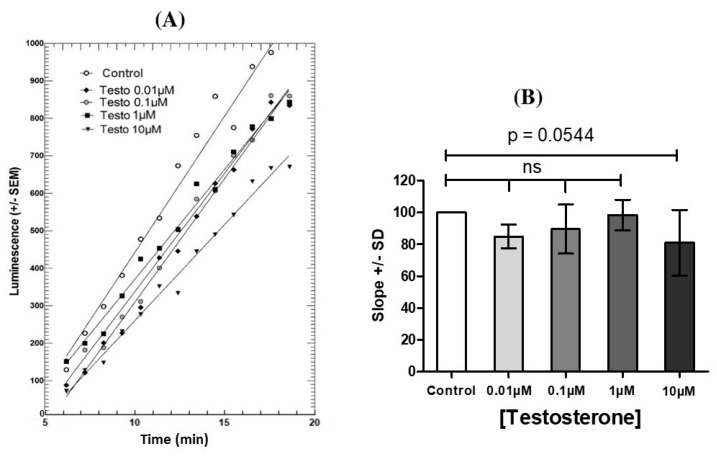
Effect of 1 h-preincubation with testosterone (Testo) alone on the cAMP accumulation in Mouse Leydig Tumor cells (mLTC)-1 cells under stimulation by 0.7 nM hLH. Left (**A**): Kinetics of oxyluciferin luminescence produced by cAMP-dependent luciferase in mLTC-1 cells stimulated by 0.7 nM rec hLH after 1h-preincubation with various concentrations of testosterone. Right (**B**): Dose-response effects from kinetics in panel (A). Each point represents the mean of luminescence in three wells in each condition. The figure presents one representative experiment out of three independent experiments. After checking for normal distribution, statistical analyses were performed by a paired Student’s *t*-test. ns = not statistically significant between each testosterone concentration and control.

Preincubation of mLTC-1 cells for 60 min with various concentrations of testosterone (Figure 1) or progesterone (not shown) had a small, but not significant, inhibitory effect on the subsequent stimulation of cAMP synthesis by 0.7 nM hLH.

**Figure 2:**
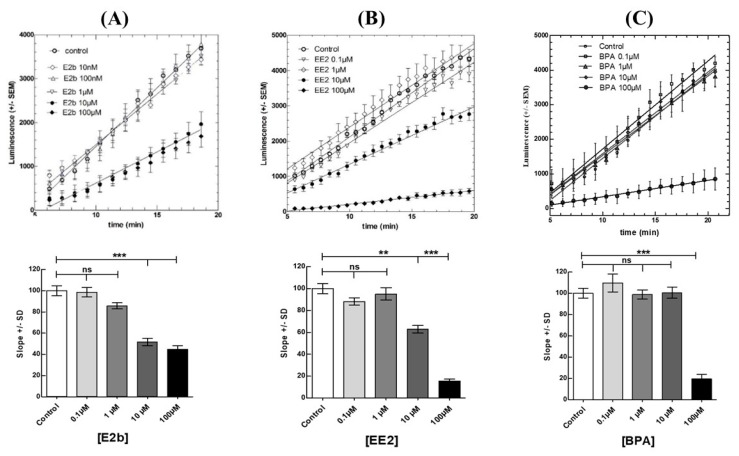
Effect of individual estrogenic molecules on the cAMP accumulation in mLTC-1 cells under stimulation by 0.7 nM hLH. Kinetics of oxyluciferin luminescence after 1h-preincubation with various concentrations of (**A**) 17β-estradiol (E2β), (**B**) ethynylestradiol (EE2), and (**C**) bisphenol-A (BPA) are shown in the upper part. The corresponding dose-related effects are shown in the lower part. Each point represents the mean of luminescence in three wells in each condition. The figure presents one representative experiment out of three independent experiments. After checking for normal distribution, the data were analyzed by a paired Student’s t-test. * indicates a significant difference (** *p* < 0.01, *** *p* < 0.001) compared between control with each concentration of E2b or EE2 or BPA, ns = not statistically significant.

In contrast to testosterone and progesterone, estrogenic molecules (E2β, EE2, and BPA) exerted a marked inhibitory effect on the subsequent stimulation by hLH (Figure 2). 17β-estradiol and EE2 at 10 µM significantly lowered the cAMP response to hLH, and BPA exerted the same effect but only at a higher concentration (100 µM).

In order to test whether the effects of estrogenic molecules could be exerted through the membrane-spanning G protein-coupled estrogen receptor (GPER) estrogen receptor, we challenged the cells with the GPER G1 agonist but found no effect on the subsequent response of the cells to hLH (data not shown). Nevertheless, we do not consider this experiment as conclusive as we have not previously tested our G1 agonist in a cell model where GPER is known to be present and active.

### 3.2. Effects of ADPN on LH-Stimulated cAMP Response

**Figure 3:**
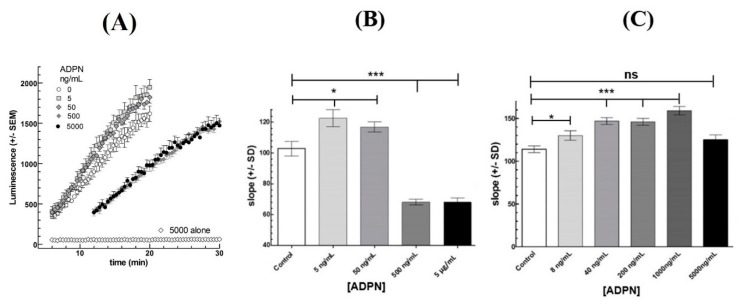
Effect of adiponectin (ADPN) on the cAMP response to 0.7 nM hLH in Mouse Leydig Tumor cells (mLTC)-1 cells. (**A**): Kinetics of oxyluciferin luminescence produced by cyclic AMP-dependent luciferase in mLTC-1 cells stimulated by 0.7 nM rec hLH after 1h-preincubation with the shown concentrations of ADPN. (**B**): Dose-response effects from kinetics in panel (A). (**C**) Dose-response effect of 1h-preincubation with frozen and thawed ADPN on the cAMP response to 0.7 nM hLH. Each point represents the mean of luminescence in six wells in each condition. The figure presents one representative experiment out of three independent experiments. After checking of normality of distribution, the data were analyzed by a paired Student’s t-test. * indicates a significant difference (* *p* < 0.05, *** *p* < 0.001) compared between each ADPN concentration and control (hLH alone), ns = not statistically significant.

Preincubation of mLTC-1 cells for 60 min with various doses of recombinant bovine ADPN had a bimodal effect on the subsequent response to 0.7 nM hLH (Figure 3B,C). At low ADPN concentration (5–125 ng/mL), an increase of up to 25% in the LH-stimulated cAMP synthesis rate was observed. At higher ADPN concentrations (0.5–5 µg/mL), a decrease of about 40% was found (Figure 3B). Interestingly, when frozen and thawed ADPN was used, the potentializing effect was observed at both low and high ADPN concentrations (Figure 3C) and was only slightly less marked at the highest concentration.

## 4. Discussion

In the present work, we have used mLTC cells, which naturally express LH receptors [27,28] and exhibit a dose-dependent cAMP response to LHs and Chorionic Gonadotropins (CGs). In previous work, we have compared the stimulation kinetics of LHs and CGs from various species in mLTC cells [29] and observed that human LH and CG exerted a strong and sustained cAMP stimulation. Recombinant hLH was thus chosen to search for possible effects of steroids and ADPN on LH-stimulated cAMP pathway in these cells. Human LH was used at 0.7 nM concentration because it is a submaximal stimulating concentration that, thus, promotes a solid cAMP response in mLTC cells but not a saturating one.

The present results clearly demonstrate that the estrogenic compounds, but not testosterone and progesterone, rapidly inhibit intracellular cAMP accumulation under hLH stimulation in mLTC-1 cells. The observation that estrogenic compounds exert their inhibitory effect in 1-h time suggests the involvement of an estrogen membrane receptor [30,31] rather than canonical nuclear receptors requiring transcriptional activity [32]. Indeed the G protein-coupled estrogen receptor (GPER) (also known as GPR30), which belongs to the GPCR family of receptors, quickly mediates the non-genomic signaling of estrogens [33]. The inhibitory mechanism of LH-stimulated cAMP accumulation by estrogens through the membrane GPER could arise through direct interaction between the two GPCRs, LHR and GPER, or at a downstream step between LHR activation and adenylate cyclase stimulation. Since LHR dimerizes upon activation by LH [34], a possible mechanism is that estrogen-occupied GPER could interfere with LHR homo-dimerization, in a way similar to the Follicle-Stimulating Hormone FSH receptor (FSHR) [35]. E2 activation of GPER leads to transactivation of the Epidermal Growth Factor Receptor (EGFR) and downstream activation of Mitogen-activated protein kinase (MAPK) and Phosphoinositide 3-kinase (PI3K) signaling cascades in breast cancer cells [36]. It is possible that in mLTC cells, one of these kinases could inhibit, by phosphorylation, the activity of the adenylate cyclase type activated downstream of LHR occupation by hLH.

It is also interesting to point out that BPA is only about 10-fold less potent than E2β and EE2 in this effect, whereas it has been previously found to be approximately 10,000-fold less efficient than these steroids in various long-term effects involving nuclear estrogen receptors [32]. This point deserves further investigation since BPA has been shown to exert some of its effects through GPER [37,38]. Nevertheless, in a preliminary experiment, we found no effect of the GPER agonist G1 on the subsequent cAMP response to LH in mLTC-1 cells. Since we did not check the activity of the G1 molecule before use in mLTC cells, we cannot conclude that this hypothesis is false and will have to study it further.

The BPA concentration, showing an effect in mLTC cells (100 µM), is considerably higher than the concentration in blood circulation (~10 nM) of old people living near electronic waste dismantling facilities [39]. Then, if BPA exerts an endocrine disruptor effect through Leydig cells steroidogenesis inhibition, it must only be after long-term exposure.

Leydig cells are targets for ADPN [40,41], and they express both the high-affinity AdipoR1 and low-affinity AdipoR2 receptors [40,42,43]. In the present work, ADPN at very low concentration was found to exert a potentializing effect on the cyclic AMP response to LH. This potentiation was observed after a 1-h preincubation of mLTC-1 cells with an ADPN concentration as low as 2 ng/mL. This concentration is more than 2000-fold lower than circulating ADPN concentrations (~4–15 µg/mL) in mammals blood [44,45]. Since ADPN has been shown to be expressed in rat Leydig cells [40], a possible autocrine role can be suggested in spite of the high circulating concentration in blood.

ADPN forms oligomers from trimer to 18-mer [46], but it is not well known which of them are the most active biologically. The commercial recombinant ADPN that we used in the present work was not characterized by this point of view. Using extemporaneously dissolved ADPN, we observed first an enhancement of cAMP accumulation at the lowest ADPN concentration and then an inhibition at higher concentrations (Figure 3B). In contrast, with frozen and thawed ADPN, we only observed the enhancement phase at low concentration but not the inhibitory one at higher concentrations (Figure 3C). It can be hypothesized that the forms of ADPN responsible for the inhibition of LH-stimulated cAMP accumulation were denatured during the freezing-thawing process as it is in redox changes [47]. The forms of ADPN responsible for these two effects might differ in their monomer numbers [48].

ADPN has been shown to act intracellularly through several pathways, and AMPK has been shown to be involved in many of its actions [49,50,51,52,53]. A previous paper reported a role for AMPK activity in mLTC-1 response to hCG [54], but it was essentially involved beyond cAMP accumulation, i.e., on steroidogenic acute regulatory protein (StAR)-promoted cholesterol entry into mitochondria and on progesterone synthesis. AMPK has been shown to be involved in another Leydig cell line (TM3) as an intermediate in NF*k*B suppression by ADPN [41]. This indicates that ADPN receptors are present in Leydig cells, and it will be interesting to test whether this hormone also modulates the steroidogenic responses to LH in testicular Leydig cells through AMPK control.

## 5. Conclusions

We report in the present paper, fast effects of both, estrogenic compounds and adiponectin, on LH-stimulated cyclic AMP accumulation in mLTC-1 cells. For the time being, the pathways are not known with sufficient precision to tackle the issue of possible cross inhibitions or synergies between them. Future studies in this direction would nevertheless be interesting to perform, considering in particular, the central role played by Peroxisome Proliferator-Activated Recptor γ PPARγ in both pathways [32,55,56].
